# Selective IgM Deficiency—An Underestimated Primary Immunodeficiency

**DOI:** 10.3389/fimmu.2017.01056

**Published:** 2017-09-05

**Authors:** Sudhir Gupta, Ankmalika Gupta

**Affiliations:** ^1^Program in Primary Immunodeficiency and Aging, Division of Basic and Clinical Immunology, University of California at Irvine, Irvine, CA, United States

**Keywords:** autoimmunity, specific antibody deficiency, CD4 Treg, CD8 Treg, Breg

## Abstract

Although selective IgM deficiency (SIGMD) was described almost five decades ago, it was largely ignored as a primary immunodeficiency. SIGMD is defined as serum IgM levels below two SD of mean with normal serum IgG and IgA. It appears to be more common than originally realized. SIGMD is observed in both children and adults. Patients with SIGMD may be asymptomatic; however, approximately 80% of patients with SIGMD present with infections with bacteria, viruses, fungi, and protozoa. There is an increased frequency of allergic and autoimmune diseases in SIGMD. A number of B cell subset abnormalities have been reported and impaired specific antibodies to *Streptococcus pneumoniae* responses are observed in more than 45% of cases. Innate immunity, T cells, T cell subsets, and T cell functions are essentially normal. The pathogenesis of SIGMD remains unclear. Mice selectively deficient in secreted IgM are also unable to control infections from bacterial, viral, and fungal pathogens, and develop autoimmunity. Immunological and clinical similarities and differences between mouse models of deficiency of secreted IgM and humans with SIGMD have been discussed. Patients with SIGMD presenting with recurrent infections and specific antibody deficiency responses appear to improve clinically on immunoglobulin therapy.

## Introduction

During ontogeny, IgM is the first immunoglobulin to be expressed on the surface of B cells (1), and the first immunoglobulin isotype secreted during an initial immune response to an exogenous antigen. Mature naïve B cells in response to antigens undergo clonal expansion and differentiation into Ig-secreting cells. In the germinal center (GC) of secondary lymphoid organs, two important events take place. A subset of activated IgM+ B cells undergo a process of heavy chain isotype switching resulting in the production of antibodies of different isotypes such as IgG, IgA, and IgE, upon engagement of CD40 with CD40L and interaction with cytokines, and somatic hypermutation in v region results in the selection of high affinity antibody producing B cells (2, 3). In contrast to secreted IgG, IgM comes in two flavors, pre-immune or without exposure to exogenous antigen also know as “natural IgM” that is spontaneously produced, and the second type is exogenous antigen-induced or “immune” IgM antibodies. The majority of circulating IgM is comprised of natural IgM antibodies. It has been established from studies of mutant mice deficient in IgM secretion that both natural and immune IgM are important in responses to pathogens and self-antigens (4, 5). The two prominent features of natural IgM are polyreactivity and autoreactivity, which are attributed to the germline configuration of their v region structures. The natural IgM antibodies are reactive with many conserved epitopes that are shared by microbes and self-antigens. The production of natural IgM appears to be triggered by interaction with self-antigens. In addition to providing early defense against microbes, natural IgM plays an important role in immune homeostasis, and provide protection from consequences of autoimmunity and inflammation (6–9). It also appears that the specificity to bind to components of self-antigen is critical for protecting effect of natural IgM against autoimmunity. In mice, B1 cell-derived plasma cells are the major source of natural IgM, and natural IgM promotes the T cell-dependent antibody response by conventional B2 cells (10). The immune IgM antibodies are selected for antigen-specificity that are usually produced in response to pathogens, and serve as a first line of defense against microbial infections and also provide protection from autoimmune diseases (4, 6).

Selective IgM Deficiency (SIGMD) is disorder with serum IgM below two standard of mean, and normal IgG, and IgA and T cell functions. In 1967, Hobbs et al. (11) first described children with SIGMD (type V dysgammaglobulinemia) presenting with meningococcal meningitis. Since then, a number of patients with SIGMD have been reported [reviewed in Ref. (12)]. There are no large-scale studies reported for the prevalence of SIGMD. In an unselected community health screening survey, a prevalence of 0.03% of “complete” SIGMD was reported (13). Recently, in screening of more than 3,000 healthy adult blood bank blood donors in Iran, the prevalence of SIGMD was 0.37% (14). A prevalence of 0.07–2.1% in Immunology and immunodeficiency clinics has been reported (15, 16).

## Genetics

Although occasional symptomatic familial cases of SIGMD deficiency have been reported (11, 17), no definitive inheritance pattern is known for SIGMD. In our immunology clinic, we are also following a mother and daughter with symptomatic SIGMD and specific antibody deficiency; both have similar clinical and immunological profile. SIGMD has been reported in a number of chromosomal abnormalities, including that of chromosome 1,18 and 22q11.2 (18–22). The most common association of SIGMD has been with 22q11.2 deletion syndrome (18–20, 23–25).

## Clinical Features

Similar to other primary immunodeficiency disorders, patients with SIGMD commonly present with recurrent infections with common microbes, and increased frequency of allergic and autoimmune diseases. These clinically features have been reviewed in detail (12). Recurrent infections as the presenting manifestation occur in more than 80% of patients with SIGMD. Some of these bacterial infections may result in serious life-threatening infections (11, 17, 21, 22). The clinical infectious presentations of SIGMD include recurrent otitis media, chronic sinusitis, bronchitis, bronchiectasis, pneumonia, urinary tract infections, cellulitis, meningitis, sepsis, etc. Some of the common microbial organisms include *Streptococcus pneumoniae, Hemophilus influenzae, Neisseria meningitidis, Pseudomonas aeruginosa, Aspergillus fumigatus, Giardia lamblia*; many of these organisms express epitopes of phosphorylcholine in their cell walls that are similar to those expressed on apoptotic cells, and recognized by natural IgM.

Although IgG levels are normal in SIGMD, increased susceptibility to infections in SIGMD may be due to associated impaired IgG-specific antibody response to T-independent polysaccharide antigens (26). These observations are analogous to mutant mice deficient in secreted IgM, in that immunoglobulin isotype switching to IgG is normal; however, these animals are also unable to control viral, bacterial, and fungal infections due to impaired induction of a protective specific IgG antibody response, and lack of serum IgM (4, 27, 28).

In children with SIGMD, allergic and autoimmune diseases are infrequent (29), whereas in adults, allergic and autoimmune diseases are frequently present (15, 26). Almost 40% of patients with SIGMD display allergic manifestations. Several investigators have reported association between atopic diseases and SIGMD (15, 26, 30, 31). The frequency of asthma and allergic rhinitis in SIGMD in reported cases ranged from 30 to 45%.

Similar to other primary antibody deficiency disorders (32), autoimmunity and autoimmune diseases (Table 1) are more common in patients with SIGMD than in the general population (33–39). Goldstein et al. (15) reported positive ANA in 13% and arthritis and SLE in 14% of patients with SIGMD. In our current cohort of 55 patients with SIGMD, we have observed Hashimoto’s thyroiditis in 8, SLE in 3, Myasthenia gravis in 2, and Addison’s disease in 1 patient (unpublished observations). Mice genetically generated to be deficient in secreted IgM spontaneously develop ANA, anti-ds-DNA, and anti-ss-DNA antibodies and autoimmune diseases like arthritis and “lupus-like” diseases (6, 40, 41). In mice deficient in secreted IgM, the prevalence of autoimmune diseases increases as mice age (6). In SIGMD, autoimmune diseases are more frequent in adults as compared to children with SIGMD (12, 15, 29). Paradoxically, levels of autoreactive IgM are elevated in patients with autoimmune diseases (42, 43). This could reflect a compensatory mechanism by which IgM inhibits inflammation and promotes clearance of apoptotic cells. Mice mutants for FcμR are impaired in IgG antibodies against T-dependent and T-independent antigens, develop autoimmunity as they age, however, have increased levels of IgM (44). Although the precise mechanism of IgM-mediated regulation of tolerance is unclear, a number of mechanisms have been proposed: (a) cross-linking of FcμR and BCR by IgM autoantibodies-self-antigen complexes resulting in the induction of anergy or deletion of B cells; (b) loss of central tolerance as a result of BCR editing at the level bone marrow; (c) loss of peripheral tolerance secondary to a deficiency of isotype-specific regulatory lymphocytes; (d) impaired clearance of apoptotic cells/bodies (self antigens). In SIGMD patients, we did not observe any significant changes in the expression of FcμR on any of B cell subsets except low-level expression on MZ B cells (45). Therefore, it is unlikely that changes in FcμR or peripheral tolerance play a significant role in the development of autoimmunity in SIGMD. Patients with SIGMD also have decreased proportions of CXCR3^+^ B cells (46). Recently, a deficiency of CXCR3^+^ B cells has been linked to autoimmune diseases (47).

**Table 1:** Autoimmune diseases associated with SIGMD.

Addison’s disease
Autoimmune glomerulonephritis
Autoimmune hemolytic anemia
Autoimmune thrombocytopenia
Celiac disease
Crohn’s disease
Hashimoto’s thyroiditis
Myasthenia gravis
Polymyositis
Rheumatoid arthritis
Sjogren’s syndrome
Systemic lupus erythematosus
Vitiligo

## Immune Responses in SIGMD

A number of abnormalities in lymphocyte numbers and functions have been reported in SIGMD. In contrast, innate immune responses appear to be preserved.

### Lymphocyte Phenotype

#### B Lymphocytes

The proportions and numbers of surface IgM^+^ B cells or CD19^+^/CD20^+^ B cells are normal in the majority of patients with SIGMD (15, 26, 48–51). However, low to complete lack of sIgM^+^, CD19^+^, or CD20^+^ B cells have been reported (23, 52–54). Recently, we have performed an extensive analysis of B cell subsets in 20 patients with SIGMD (46). A significant increase in CD21^low^, and IgM memory B cells, and a significant decreased in GC B cells, and CXCR3^+^ naïve and memory B cells were observed in SIGMD. Lau et al. (55) have demonstrated that CD21^low^ cells are recent GC graduates that represent a distinct population from CD27^+^ memory cells, are refractory to GC reentry, and are predisposed to differentiate into long-lived plasma cells. Therefore, a deficiency of CD21^low^ cells may be responsible for decreased differentiation to plasma cells and decreased Ig production. However, it cannot explain defects in selective production of IgM. CD21^low^ are increased in patients with CVID with autoimmunity, and systemic lupus erythematosus (53, 54, 56). Impaired polysaccharide response early life is believed to be secondary to low expression of CD21 on B cells. A significant increase in the proportions of CD21^low^ B cells in our patients with SIGMD may explain both increased autoimmunity and impaired anti-polysaccharide antibody responses in SIGMD.

A role of CXCR3 in T cells and T-cell-mediated autoimmunity has been well established (57); however, its role in B cell trafficking and antibody-mediated autoimmunity is beginning to emerge (58). Patients with SLE have decreased number of CXCR3^+^ B cells (45). We have observed decreased expression of CXCR3 on both naïve and memory B cells in SIGMD (46). A role of decreased expression of CXCR3 on B cell subsets in SIGMD and autoimmunity associated with SIGMD remains to be investigated.

#### T Lymphocytes

Number and functions of T cell and T cell subsets are normal in the majority of patients with SIGMD (26, 29, 46, 48–50, 59) except in the syndrome of SIGMD, T cell deficiency, and MAC infection (12, 60, 61).

Upon antigenic stimulation, naïve CD4^+^ and CD8^+^ T cells undergo clonal expansion, differentiation, and distinct homing patterns. Based upon their functions and homing patterns, both CD4^+^ and CD8^+^ T cells have been classified into naïve (T_N_), central memory (T_CM_), effector memory (T_EM_), and terminally differentiated effector memory (T_EMRA_) subsets (62–64). Patients with SIGMD are reported to have normal distribution of T_N_, T_CM_, T_EM_, and T_EMRA_ subsets of CD4^+^ and CD8^+^ T (46, 50). In contrast, T_N_ and T_CM_ subsets of both CD4^+^ and CD8^+^ T cells were decreased, whereas T_EMRA_ subset of CD4^+^ and CD8^+^ T cells were markedly increased in patients with syndrome of SIGMD, T cell deficiency, and MAC infection (60, 61).

#### Regulatory Lymphocytes

Although a role of CD4^+^ Treg in immune tolerance is well established (65), it is only recently there has been an interest in the role of Breg (66) and CD8Treg (67, 68) in immune homeostasis, and tolerance.

Breg have shown to regulate various immune responses including regulation of generation of CD4^+^ Treg; deficiency of Breg results in experimental autoimmune diseases, and more recently, Breg were shown to regulate the generation of peripheral CD4^+^ Treg cells (66, 69–72). We have reported significant increase in Breg in patients with SIGMD (46). It is unclear whether increase in Breg and CD8^+^ Treg (see below) are compensatory changes to control the development of autoimmunity and autoimmune diseases in SIGMD, or they contribute to disease phenotype. If increased Breg contribute to SIGMD by suppressing directly B cell differentiation to Ig-secreting plasma cells remains to be investigated.

A role of CD8 Treg cells in immune homeostasis has been demonstrated in a number of experimental models and their alterations have been observed in human diseases (67, 68, 73–75). In our cohort of patients with SIGMD, CD8 Treg cells (CD8^+^CCR7^+^CD183^+^CD45RA^−^) were increased (46).

### Lymphocyte Proliferative Response

The lymphocyte transformation in response to mitogens phytohemagglutinin, concanavalin A, and pokeweed mitogen, recall antigens, *Candida albicans*, mumps, and tetanus toxoid, and alloantigens appears to be intact in SIGMD (26, 48), demonstrating normal functioning T cells. Yamasaki (48) observed impaired B cell proliferation in six patients with SIGMD in response to *Staphylococcus aureus* Cowan (SAC) strain I (a B cell activator), suggesting B cell functional defect in SIGMD. Mensen et al. (50) examined the expansion of B cells and antibody secreting cells following stimulation of peripheral blood mononuclear cell (PBMC) or non-T cells with a cocktail of B cell stimuli. A decreased expansion/survival of all subsets of B cells in five of six patients, and decreased IgM secreting B cells in two of six patients were observed.

### Serum Immunoglobulins and Specific Antibody Responses

#### Serum Immunoglobulins

By definition, SIGMD is characterized by serum IgM levels below 2 SD of mean for a laboratory with normal serum IgG and IgA. However, patients with complete absence (≤3 mg/dl) of serum IgM have been reported in SIGMD. In our cohort of 55 patients with SIGMD, 4 patients have complete absence of serum IgM. IgG subclass deficiency has been reported in few cases of SIGMD (26, 76). IgG subclass deficiency is not restricted to any particular subclass. This would be analogous to association of IgG subclass deficiency with selective IgA deficiency. Because of small cohort of cases, it is difficult to determine the clinical significance of IgG subclass deficiency in patients with SIGMD. Patients with SIGMD have increased prevalence of allergic diseases, therefore, it is not surprising that serum IgE levels are increased in a subset of SIGMD (26, 48, 59, 76, 77).

#### Specific Antibody Responses

Specific antibody responses to T-dependent protein antigens (e.g., tetanus, diphtheria), and T-independent antigens (anti-polysaccharide antigens) have been studied in a small number of patients with SIGMD. Antibody response to diphtheria and tetanus in a small number of patients with SIGMD studied are reported to be normal (25, 26), except impaired response in the syndrome of SIGMD, T cell deficiency, and mycobacterial infection (60, 61). A role of specific antibodies in mycobacterial defense is discussed (61). The response to T-independent antigens appeared to be impaired (18, 22, 25, 26, 78). We reported impaired antibody responses against *S. pneumoniae* following Pneumovax-23 vaccination in 45% of SIGMD (26). In our current cohort of 55 patients with SIGMD, more than 45% have impaired anti-pneumococcal antibody responses (unpublished observations). Al-Herz et al. (18) and Guill et al. (30) reported decreased IgM Isohemagglutinins titers in a subset of patients with SIGMD.

## Pathogenesis of SIGMD

The pathogenesis of SIGMD remains unclear. A number of mechanisms have been proposed (Table 2). Since the majority of patients have normal surface IgM^+^ B cells, investigators have focused their studies on the analysis of helper T cells, regulatory (suppressor) cells, and intrinsic defects of B cells. A deficiency of helper T cells (59), increased IgM isotype-specific suppressor T cells (49, 79–81), intrinsic B cell defects (48, 52), and reduced secreted μ mRNA transcripts (82) have been reported as possible pathogenic mechanisms for SIGMD. De la Concha et al. (59) reported that B cells from SIGMD patients could produce normal amounts of IgM, IgG, and IgA when coculture *in vitro* with T cells from healthy controls, suggesting a defect in T-helper cell function. However, this defect was not IgM isotype specific. In contrast, Matsushita et al. (80) reported the presence of radiosensitive IgM-specific suppressor T cells in a patient with SIGMD and giant leiomyoma of the stomach. Inoue et al. (79) using *in vitro* cocultures revealed an increased IgM isotype-specific suppressor T cell activity in all seven patients with SIGMD. Ohno et al. (49), in a recombination plaque assay, observed increased isotype IgM-specific (one case) and non-specific (one case) suppressor activity in patients with SIGMD. We have observed that CD8 Treg *in vitro* suppress immunoglobulin secretion by purified B cells (unpublished personal observations); therefore, increased CD8^+^ Treg in SIDMD (46) may play a role in the pathogenesis of SIGMD.

**Table 2:** Proposed mechanisms for the pathogenesis of SIGMD.

Mechanisms	Reference
Decreased T helper cell activity	(59)
Increased isotype specific suppressor T cells	(49, 79, 80)
Increased CD8 Treg cells	(46)
Intrinsic B cell defect	(48, 52)
Increased Breg	(46)
Defective secretion of μ mRNA transcripts	(82)
^a^Mutations in protein transport gene(s)	

*^a^Proposed by authors*.

In contrast, Karsh et al. (52) failed to observe any defect in T helper or T suppressor activity in SIGMD and suggested a possible intrinsic B cell defect. Takeuchi and associates (33) demonstrated that *in vitro* stimulation of PBMCs from a patient with SIGMD and systemic lupus erythematosus with IL-2 and SAC (a B cell activator) did not increased IgM synthesis, suggesting an intrinsic B cell defect. Furthermore, sequence analysis of μ heavy chain, and the IgM mRNA did not reveal any mutation or deletion. Mensen et al. (50), in a T-dependent B cell activation experimental system and using ELISA spot assay, observed decreased number of IgM-secreting cells in two of six patients with SIGMD as compared to healthy controls. Yamasaki (48) analyzed T and B cells from six patients with SIGMD. Purified B cells from SIGMD patients following *in vitro* stimulation with *SAC* in the presence of T cells from healthy controls secreted decreased amounts of IgM as compared to B cells from healthy controls. Furthermore, stimulation of patient’s B cells with SAC and B cell differentiation factor (BCDF) did not increase IgM secretion. These data suggest an intrinsic B cell defect, and not of helper T cell defect or impaired BCDF production in SIGMD. We have observed increased proportion of Breg in SIGMD (46).

Whether increased Breg contribute to SIGMD by suppressing directly B cell differentiation to Ig-secreting plasma cells remains to be investigated. Since membrane bound IgM^+^ B cells are normal, we propose there is a likelihood of gene defect(s) responsible for transport of secreted proteins, including IgM.

## Immunoglobulin Therapy

The replacement of IgM would be an ideal therapy for SIGMD patient. Hurez et al. (83) reported that passive transfer of IgM enriched (90%) preparation from normal human donors, in a dose-dependent manner, inhibited *in vitro* binding of a variety of autoantibodies to target autoantigens, and *in vivo* prevented the onset of experimental autoimmune uveitis in rats. Warrington et al. (84) demonstrated that the administration of recombinant human monoclonal IgM that binds to oligodendrocytes-induced remyelination in mice with chronic virus-induced demyelination, a model of chronic progressive multiple sclerosis. Vassilev and colleagues (85) demonstrated that the administration of highly enriched IgM (more than 90%) from pooled plasma of healthy donors inhibited experimental myasthenia gravis in SCID mice model that was associated with marked decreased in anti-choline receptor antibodies. However, there are no commercial immunoglobulin preparations available that is highly enriched in IgM. Since more than 45% of patients with SIGMD have an impaired specific anti-pneumococcal IgG antibody response (a defect also observed in mice with mutations in IgM FcμR), current immunoglobulin preparations may be beneficial in this subsets of clinically symptomatic patients with SIGMD. Yel et al. (26) reported favorable response to intravenous immunoglobulin in SIGMD patients with regard to frequency and severity of infections. Stoelinga et al. (35) and Fallon (86) also observed beneficial effects of IVIG in SIGMD. Goldstein and associates (87), in a retrospective chart analysis, observed clinical improvement in four patients with a triad of SIGMD, bronchiectasis, and asthma using high dose IVIG. However, in these patients no information is provided for specific antibody responses. Recently, Patel and colleagues (78) reported a patient with SIGMD and non-protective pneumococcal antibody titers, and recurrent multiple infections who responded to subcutaneous immunoglobulin therapy. Therefore, symptomatic SIGMD patients with specific antibody deficiency may be considered candidates for immunoglobulin treatment.

## Summary

Selective IgM deficiency is more common than previously recognized and is likely a heterogeneous disorder. Patients with SIGMD may be asymptomatic; however, commonly present with chronic and recurrent infections; some of them could be serious and life threatening. Interestingly, patients with common variable immunodeficiency with low serum IgM are clinically worse than those with normal IgM levels. There is an increased frequency of allergic and autoimmune manifestations in SIGMD. Autoimmunity in SIGMD and in mice deficient in secreted IgM and FcμR mutations, and the notably prevention of experimental autoimmune diseases by IgM supports an important immunomodulatory role of IgM. The genetic basis of SIGMD is currently unclear; however, whole exome sequencing, GWAS, and NGS may reveal gene(s) responsible for SIGMD. Patients with SIGMD should undergo a thorough immunological evaluation, particularly with history of recurrent/severe infections. A definitive diagnosis of SIGMD, especially in children, should be established after follow-up for months to a year as in few undocumented cases serum IgM levels have normalized, and after excluding any known cause of secondary SIGMD including drug (e.g., Clozapine) induced IgM deficiency (88). Furthermore, immunoglobulin treatment is a beneficial therapeutic approach for symptomatic patients with SIGMD who also have specific IgG antibody deficiency. Finally, highly enriched IgM preparations may be most desirable therapeutic modality for SIGMD with possible antimicrobial, and immunomodulatory effects on autoimmune manifestations of SIGMD.

## Author Contributions

AG collected and reviewed published material and wrote first draft. SG edited and proof read the manuscript.

## Conflict of Interest Statement

The authors declare that the research was conducted in the absence of any commercial or financial relationships that could be construed as a potential conflict of interest.

## References

[1] Gupta S Pahwa R O’Reilly R Good RA Siegal FP. Ontogeny of lymphocyte subpopulations in human fetal liver. Proc Natl Acad Sci U S A (1976) 73:919–22. 10.1073/pnas.73.3.919 1062806 PMC336031

[2] Honjo T Kinoshita K Muramatsu M. Molecular mechanism of class switch recombination: linkage with somatic hypermutation. Annu Rev Immunol (2002) 20:165–96. 10.1146/annurev.immunol.20.090501.112049 11861601

[3] Manis JP Tian M Alt FW. Mechanism and control of class-switch recombination. Trends Immunol (2002) 23:31–9. 10.1016/S1471-4906(01)02111-1 11801452

[4] Boes M Prodeus AP Schmidt T Caroll MC Chen J Critical role of immunoglobulin M in immediate defense against systemic bacterial infection. J Exp Med (1998) 188:2381–6. 10.1084/jem.188.12.2381 9858525 PMC2212438

[5] Nguyen TTT Baumgarth N Natural IgM and development of B cell-mediated autoimmune diseases. Crit Rev Immunol (2016) 36:163–77. 10.1615/CritRevImmunol.2016018175 27910766 PMC5292326

[6] Boes M Schmidt T Linkemann K Beaudette BC Marshak-Rothstein A Chen J Accelerated development of IgG autoantibodies and autoimmune diseases in absence of secreted IgM. Proc Natl Acad Sci U S A (2000) 97:1184–9. 10.1073/pnas.97.3.1184 10655505 PMC15562

[7] Mannoor K Xu Y Chen C Natural autoantibodies and associated B cells in immunity and autoimmunity. Autoimmunity (2013) 46:138–47. 10.3109/08916934.2012.748753 23186367

[8] Boes M Esau C Fischer MB Schmidt T Carroll M Chen J. Enhanced B-1 cell development, but impaired IgG antibody responses in mice deficient in secreted IgM. J Immunol (1998) 160:4776–87. 9590224

[9] Ehrenstein MR Notley CA. The importance of natural IgM: scavenger, protector and regulator. Nat Rev Immunol (2010) 10:778–86. 10.1038/nri2849 20948548

[10] Savage HP Baumgarth N Characteristics of natural antibody secreting cells. Ann N Y Acad Sci (2015) 1352:132–42. 10.1111/nyas.12799 PMC4679694 26104151

[11] Hobbs JR Milner RD Watt PJ Gamma-M deficiency predisposing to meningococcal septicaemia. Br Med J (1967) 4:583–6. 10.1136/bmj.4.5579.583 4168495 PMC1749295

[12] Louis AG Gupta S Selective IgM deficiency: an ignored primary immunodeficiency disease. Clin Rev Allergy Immunol (2014) 46:104–11. 10.1007/s12016-013-8375-x 23760686

[13] Cassidy JT Nordby GL. Human serum immunoglobulin concentrations: prevalence of immunoglobulin deficiencies. J Allergy Clin Immunol (1975) 55:35–48. 10.1016/S0091-6749(75)80006-6 803275

[14] Entezari N Adab Z Zeydi M Saghafi S Jamali M Kardar GA The prevalence of selective immunoglobulin M deficiency (SiGMD) in Iranian volunteer blood donors. Hum Immunol (2016) 77:7–11. 10.1016/j.humimm.2015.09.051 26429316

[15] Goldstein MF Goldstein AL Dunsky EH Dvorin DJ Belecanech GA Shamir K Selective IgM immunodeficiency: retrospective analysis of 36 adult patients with review of literature. Ann Allergy Asthma Immunol (2006) 97:717–30. 10.1016/S1081-1206(10)60962-3 17201230

[16] Kutukculer N Gulez N Outcome of patients with unclassified hypogammaglobulinemia in early childhood. Paeditr Allergy Immunol (2009) 20:693–8. 10.1111/j.1399-3038.2008.00845.x 19196447

[17] Jones DM Tobin BM Butterworth A Three cases of meningococcal infection in a family associated with a deficient immune response. Arch Dis Child (1973) 48:742–3. 10.1136/adc.48.9.742 4733645 PMC1648490

[18] Al-Herz W McGeady SJ Gripp KW 22q11.2 deletion syndrome and selective IgM deficiency: an association of a common chromosome abnormalities with a rare immunodeficiency. Am J Med Genet (2004) 127:99–100. 10.1002/ajmg.a.20639 15103727

[19] Gennery AR Barge D O’Sullivan JJ Flood TJ Abinun M Cant AJ Antibody deficiency and autoimmunity in 22q11.2 deletion syndrome. Arch Dis Child (2004) 86:422–5. 10.1136/adc.86.6.422 PMC1763000 12023174

[20] Shubert MS Moss RB. Selective polysaccharide antibody deficiency in familial DiGeorge syndrome. Ann Allergy (1992) 69:231–8. 1524280

[21] Zaka-ur-Rab Z Gupta P *Psuedomonas* septicemia in selective IgM deficiency. Indian Pediatr (2005) 42:961–2. 16208067

[22] Hong R Gupta S Selective IgM deficiency in an adult with *Streptococcus pneumoniea* sepsis and invasive aspergillosis. J Investig Allergol Clin Immunol (2008) 18:214–8. 18564634

[23] Haddad ZH Allen RF Towner JW Wilson MG IgA, IgM and partial deletion of chromosome 18. Lancet (1969) 1:678 10.1016/S0140-6736(69)92044-3 4179912

[24] Ostergaard PA A girl with recurrent infections, low IgM, and an abnormal chromosome number 1. Acta Paediatr Scand (1973) 62:211–5. 10.1111/j.1651-2227.1973.tb08095.x 4570826

[25] Kung S-J Gripp KW Stephen MJ Fairchok MP McGeady SJ Selective gM deficiency and 22q11.2 deletion syndrome. Ann Allergy Asthma Immunol (2007) 99:87–92. 10.1016/S1081-1206(10)60627-8 17650836

[26] Yel L Ramanuja S Gupta S. Clinical and immunological features in IgM deficiency. Int Arch Allergy Immunol (2009) 150:291–8. 10.1159/000222682 19494527

[27] Baumgarth N Herman OC Jager GC Brown LE Herzenberg LA Chen J B1- and B-2 cell-derived immunoglobulin M antibodies are nonredundant components of the protective response to influenza virus infection. J Exp Med (2000) 192:271–80. 10.1084/jem.192.2.271 10899913 PMC2193249

[28] Subramaniam KS Datta K Quintero E Manix C Marks MS Pirofski LA. The absence of serum IgM enhances the susceptibility of mice to pulmonary challenge with *Cryptococcus neoformans*. J Immunol (2010) 184:5755–67. 10.4049/jimmunol.0901638 20404271 PMC2885446

[29] Goldstein MF Goldstein AL Dunsky EH Dvorin DJ Belecanech GA Shamir K Pediatric selective IgM deficiency. Clin Dev Immunol (2008) 2008:624850 10.1155/2008/624850 19043622 PMC2587688

[30] Guill MF Brown DA Ochs HD Pyun K Moffitt J IgM deficiency: clinical spectrum and immunological assessment. Ann Allergy (1989) 62:547–52. 2735562

[31] Kaufman HS Hobbs JR Immunoglobulin deficiencies in an atopic population. Lancet (1970) ii:1061–3. 10.1016/S0140-6736(70)90288-6 4098353

[32] Gupta S Louis AG Tolerance and autoimmunity in primary immunodeficiency diseases. Clin Rev Allergy Immunol (2013) 45:162–9. 10.1007/s12016-012-8345-8 23296947

[33] Takeuchi T Nakagawa T Maeda Y Hirano S Sasaki-Hayashi M Makino S Functional defect of B lymphocytes in a patient with selective IgM deficiency associated with systemic lupus erythematosus. Autoimmunity (2001) 34:115–22. 10.3109/08916930109001959 11905841

[34] Bandilla KK Pitts NC McDuffie FC Immunoglobulin M deficiency in the immune response of patients with rheumatoid arthritis. Arthritis Rheum (1970) 13:214–21. 10.1002/art.1780130302 5310458

[35] Stoelinga GBA van Munster PJJ Slooff JP Antibody deficiency syndrome and autoimmune hemolytic anemia in a boy with isolated IgM deficiency dysgammaglobulinemia type 5. Acta Paediatr Scand (1969) 58:352–62. 10.1111/j.1651-2227.1969.tb04731.x 4188208

[36] Antar M Lamarche J Peguero A Reiss A Cole S A case of selective IgM deficiency and autoimmune glomerulonephritis. Clin Exp Nephrol (2008) 12:300–4. 10.1007/s10157-008-0049-2 18365138

[37] Shigeru K Mari T Yasumitsu N IgM deficiency in a patient with Hashimoto’s thyroiditis. Intern Med (1993) 2:302–7. 10.2169/internalmedicine.32.302 8358120

[38] Hobbs JR Hepner GW Deficiency of γM-globulin in celiac disease. Lancet (1968) 2:217–20. 10.1016/S0140-6736(68)90846-5

[39] Sugita K Eguchi M Chronic idiopathic thrombocytic purpura in a young male with isolated IgM deficiency. Int J Hematol (2001) 73:532–3. 10.1007/BF02994018 11503970

[40] Ehrenstein MR Cook HT Neuberger MS. Deficiency in serum immunoglobulin (Ig) M predisposes to development of IgG autoantibodies. J Exp Med (2000) 191:1253–8. 10.1084/jem.191.7.1253 10748243 PMC2193170

[41] Notley CA Brown MA Wright GP Ehrenstein MR. Natural IgM is required for suppression of inflammatory arthritis by apoptotic cells. J Immunol (2011) 186:4967–72. 10.4049/jimmunol.1003021 21383247

[42] Witte T IgM antibodies against dsDNA in SLE. Clin Rev Allergy Immunol (2008) 34:345–7. 10.1007/s12016-007-8046-x 18097774

[43] Cronwall C Vas J Silverman GJ Protective role of natural IgM antibodies. Front Immunol (2012) 3:66 10.3389/fimmu.2012.00066 22566947 PMC3341951

[44] Ochida R Mori H Takatsu H Kurosaki T Tokuhisha T Ohno H. Critical role of the IgMFc receptor in IgM homeostasis, B-cell survival, and humoral immune responses. Proc Natl Acad Sci U S A (2012) 109:E2699–706. 10.1073/pnas.1210706109 22988094 PMC3479561

[45] Gupta S Agrawal S Gollapudi S Kubagawa H FcµR in human B cell subsets in primary selective IgM deficiency, and regulation of FcµR and production of natural IgM antibodies by IGIV. Hum Immunol (2016) 77:1194–2001. 10.1016/j.humimm.2016.10.003 27751883 PMC5812685

[46] Louis AG Agrawal S GUPTA S Analysis of B cell subsets, Breg, CD4 Treg and CD8 Treg in adult patients with primary selective IgM deficiency. Am J Clin Exp Immunol (2016) 5:21–32. 27168952 PMC4858603

[47] Henneken M Dorner T Burmester GR Berek C. Differential expression of chemokine receptors on peripheral blood B cells from patients with rheumatoid arthritis, and systemic lupus erythematosus. Arth Res Ther (2005) 7:R1001–13. 10.1186/ar1776 16207316 PMC1257429

[48] Yamasaki T. Selective IgM deficiency: functional assessment of peripheral blood lymphocytes *in vitro*. Int Med (1992) 31:866–70. 10.2169/internalmedicine.31.866 1450494

[49] Ohno T Inaba M Kuribayashi K Masuda T Kanoh T Uchino H. Selective IgM deficiency in adults: phenotypically and functionally altered profiles of peripheral blood lymphocytes. Clin Exp Immunol (1987) 68:630–7. 2958191 PMC1542763

[50] Mensen A Krause T Hanitsch LG Meisel C Kleint ME Volk HD Altered B-cell subsets and functional B-cell defects in selective IgM deficiency. Clin Immunol (2015) 161:96–102. 10.1016/j.clim.2015.08.017 26342539

[51] Ross IN Thompson RA. Severe selective IgM deficiency. J Clin Pathol (1976) 29:773–7. 10.1136/jcp.29.9.773 977778 PMC476177

[52] Karsh J Watts CG Osterland CK Selective immunoglobulin M deficiency in an adult: assessment of immunoglobulin production by peripheral blood lymphocytes *in vitro*. Clin Immunol Immunopathol (1982) 25:386–94. 10.1016/0090-1229(82)90203-3 6891628

[53] Arumugakani G Wood PMD Carter CRD Frequency of Treg is reduced in CVID patients with autoimmunity and splenomegaly and is associated with expanded CD21^lo^ B lymphocytes. J Clin Immunol (2010) 30:292–300. 10.1007/s10875-009-9351-3 19997968

[54] Wehr C Eibel H Masilamani M Illges H Sclesier M Peter HH A new CD21^lo^ B cell population in peripheral blood of patients with SLE. Clin Immunol (2004) 113:161–71. 10.1016/j.clim.2004.05.010 15451473

[55] Lau D Lan LY-L Andrews SF Henry C Rojas KT Neu KE Low CD21 expression defines a population of recent germinal center graduates primed for plasma cell differentiation. Sci Immunol (2017) 2:aai8153. 10.1126/sciimmunol.aai8153 28783670 PMC5896567

[56] Rakhmanov M Keller B Gutenberger S Foerster C Hoenig M Driessen G Circulating CD21^lo^ B cells in common variable immunodeficiency resemble tissue homing, innate-like B cells. Proc Natl Acad Sci U S A (2009) 106:13451–6. 10.1073/pnas.0901984106 19666505 PMC2726348

[57] Antonelli A Ferrari SM Giuggioli D Ferrannini E Ferri C Fallahi P Chemokine (C-X-C motif) ligand (CXCL) 10 in autoimmune diseases. Autoimmun Rev (2014) 13:272–80. 10.1016/j.autrev.2013.10.010 24189283

[58] Lacotte S Brun S Muller S Dumortier H. CXCR3, inflammation, and autoimmune diseases. Ann N Y Acad Sci (2009) 1173:310–7. 10.1111/j.1749-6632.2009.04813.x 19758167

[59] De la Concha EG Garcia-Rodriguez MC Zabay JM Laso MT Alonso F Bootello A Functional assessment of T and B lymphocytes in patients with selective IgM deficiency. Clin Exp Immunol (1982) 49:670–6. 6983403 PMC1536738

[60] Gupta S Agrawal S Gollapudi S Selective IgM deficiency with severe T cell deficiency and *Mycobacterium avium complex* (MAC) infection. Open J Immunol (2012) 5:8–12. 10.2174/1874226201205010008

[61] Gharib A Louis AG Agrawal S Gupta S. Syndrome of selective IgM deficiency with severe T cell deficiency associated with disseminated cutaneous *Mycobacterium avium intracellulaire* infection. Am J Clin Exp Immunol (2015) 4:15–27. 26550546 PMC4620120

[62] Sallusto F Geginat J Lanzavecchia A. Central memory and effector memory T cell subsets: function, generation, and maintenance. Annu Rev Immunol (2004) 22:745–63. 10.1146/annurev.immunol.22.012703.104702 15032595

[63] Gupta S Bi R Su K Yel L Chiplunkar S Gollapudi S. Characterization of naïve, memory, and effector CD8+ T cells: effect of age. Exp Gerontol (2004) 39:545–50. 10.1016/j.exger.2003.08.013 15050289

[64] Gupta S. Molecular mechanisms of apoptosis in the cells of the immune system in human aging. Immunol Rev (2005) 205:114–29. 10.1111/j.0105-2896.2005.00261.x 15882349

[65] Buckner JH. Mechanisms of impaired regulation by CD4(+)CD25(+)FOXP3(+) regulatory T cells in human autoimmune diseases. Nat Rev Immunol (2010) 10:849–59. 10.1038/nri2889 21107346 PMC3046807

[66] Mauri C Bosma A. Immune regulatory function of B cells. Annu Rev Immunol (2012) 30:221–41. 10.1146/annurev-immunol-020711-074934 22224776

[67] Vuddamalay Y van Meerwijk JPM. CD28- and CD28^low^ CD8^+^ regulatory T cells: of mice and men. Front Immunol (2017) 8:1–7. 10.3389/fimmu.2017.00031 28167946 PMC5256148

[68] Shi Z Okuno Y Rifa’i M Endharti AT Akane K Isobe K Human CD8+CXCR3+ T cells have the same function as murine CD8+CD122+ Treg. Eur J Immunol (2009) 39:2106–19. 10.1002/eji.200939314 19609979

[69] Mauri C Blair PA. Regulatory B cells in autoimmunity: developments and controversies. Nat Rev Rheumatol (2010) 6:636–43. 10.1038/nrrheum.2010.140 20856268

[70] Mizoguchi A Bhan AK. A case for regulatory B cells. J Immunol (2006) 176:705–10. 10.4049/jimmunol.176.2.705 16393950

[71] Flores-Borja F Bosma A Ng D Reddy V Ehrenstein MR Isenberg DA CD19+CD24hiCD38hi B cells maintain regulatory T cells while limiting TH1 and TH17 differentiation. Sci Transl Med (2013) 5:173ra123. 10.1126/scitranslmed.3005407 23427243

[72] Wei B Velazquez P Turovskaya O Spricher K Aranda R Kronenberg M Mesenteric B cells centrally inhibit CD4+ T cell colitis through interaction with regulatory T cell subsets. Proc Natl Acad Sci U S A (2005) 102:2010–5. 10.1073/pnas.0409449102 15684084 PMC548553

[73] Endharti AT Okuno Y Shi Z Misawa N Toyokuni S Ito M CD8+CD122+ regulatory T cells (Tregs) and CD4+ Tregs cooperatively prevent and cure CD4+ cell-induced colitis. J Immunol (2011) 186:41–52. 10.4049/jimmunol.1000800 21098236

[74] Lee YH Ishida Y Rifa’i M Shi Z Isobe K Suzuki H. Essential role of CD8+CD122+ regulatory T cells in the recovery from experimental autoimmune encephalomyelitis. J Immunol (2008) 180:825–32. 10.4049/jimmunol.180.2.825 18178821

[75] Tennakoon DK Mehta RS Ortega SB Bhoj V Racke MK Karandikar NJ. Therapeutic induction of regulatory, cytotoxic CD8+ T cells in multiple sclerosis. J Immunol (2006) 176:7119–29. 10.4049/jimmunol.176.11.7119 16709875

[76] Ideura G Agematsu K Komatsu Y Hatayama O Yasuo M Tsushima K Selective IgM deficiency accompanied with IgG4 deficiency, dermal complications and a bronchial polyp. Allegol Int (2008) 7:99–105. 10.2332/allergolint.C-06-52 18089938

[77] Yocum MW Strong D Chusid MJ Lakin JD. Selective immunoglobulin M (IgM) deficiency in two immunodeficient adults with recurrent staphylococcal pyoderma. Am J Med (1976) 60:486–94. 10.1016/0002-9343(76)90714-2 1274982

[78] Patel SS Fergeson JE Glaum MC Lockley RF Symptomatic primary selective IgM deficiency with nonprotective pneumococcal titers responsive to subcutaneous immunoglobulin. Int Arch Allergy Immunol (2016) 170:138–40. 10.1159/000447693 27505292

[79] Inoue T Okumura Y Shirahama M Ishibashi H Kashiwagi S Okuba H. Selective partial IgM deficiency: functional assessment of T and B lymphocytes *in vitro*. J Clin Immunol (1986) 6:130–5. 10.1007/BF00918745 2872228

[80] Matsushita S Inoue T Okubo H. A case of selective IgM deficiency: isotype-specific suppressor T lymphocytes. Jpn J Med (1984) 23:149–51. 10.2169/internalmedicine1962.23.149 6233438

[81] Raziuddin S Bilal N Benjamin B. Transient T-cell abnormality in a selective IgM-immunodeficient patient with *Brucella* infection. Clin Immunol Immunopathol (1988) 46(3):60–367. 10.1016/0090-1229(88)90055-4 2962796

[82] Kondo N Ozawa T Kato Y Motoyoshi F Kashahara K Kameyama T Reduced secreted mu mRNA synthesis in selective IgM deficiency of Bloom’s syndrome. Clin Exp Immunol (1992) 88:35–40. 10.1111/j.1365-2249.1992.tb03035.x 1563106 PMC1554351

[83] Hurez V Kazatchkine MD Vassilev T Ramanathan S Pashov A Basuyaux B Pooled normal human polyspecific IgM contains neutralizing anti-idiotypes to IgG autoantibodies of autoimmune patients and protects from experimental autoimmune disease. Blood (1997) 90:4004–13. 9354669

[84] Warrington AE Bieber AJ Ciric B Pease LR Van Keulen V Rodriguez M. A recombinant human IgM promotes myelin repair after a single, very low dose. J Neurosci Res (2007) 85:967–76. 10.1002/jnr.21217 17304578

[85] Vassilev T Yamamoto M Aissaoui A Bonnin E Berrih-Aknin S Kazatchkine MD Normal human immunoglobulin suppresses experimental myasthenia gravis in SCID mice. Eur J Immunol (1999) 29:2436–42. 10.1002/(SICI)1521-4141(199908)29:08<2436::AID-IMMU2436>3.3.CO;2-0 10458757

[86] Fallon KE Inability to train, recurrent infection, and selective IgM deficiency. Clin J Sport Med (2004) 14:357–9. 10.1097/00042752-200411000-00006 15523208

[87] Goldstein MF Hildich GJ Dvorin DJ Belecanech GA Immunoglobulin replacement for selective IgM deficiency, bronchiectasis, and asthma. Ann Allergy Asthma Immunol (2016) 116:172–3. 10.1016/j.anai.2015.11.017 26712522

[88] Lozano R Marin R Pascual A. Selective immunoglobulin M deficiency among clozapine-treated patients: a nested case-control study. Prim Care Companion CNS Disord (2015) 17(4). 10.4088/PCC.15m01782 26693046 PMC4664574

